# Supplementary figures and images for: Vision-related convergent gene losses reveal SERPINE3’s unknown role in the eye
Source: eLife. 2022 Jun 21;11:e77999. doi: 10.7554/eLife.77999 (PMC9355568; doi:10.7554/eLife.77999)

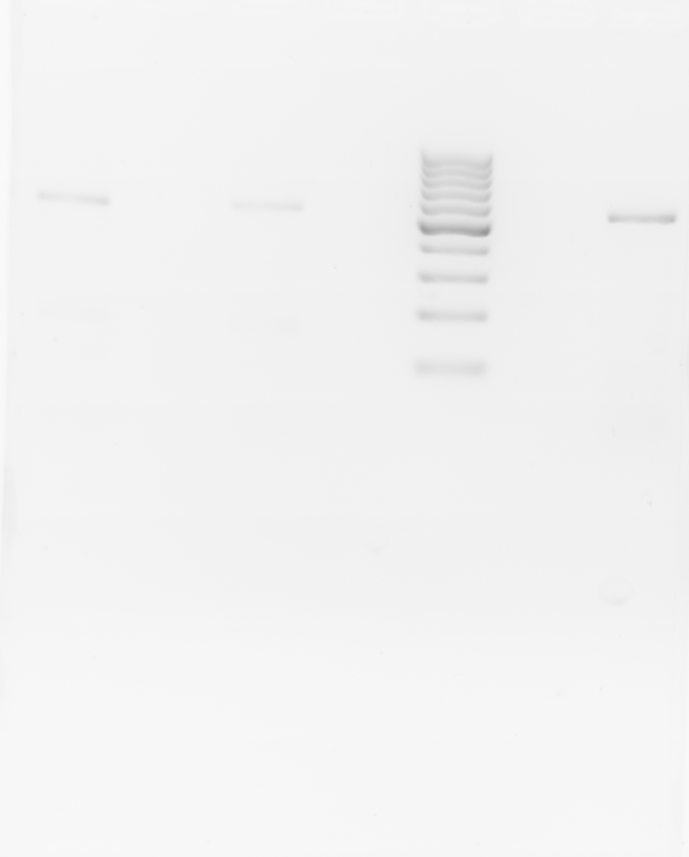

Supplement: Figure 4—source data 4. [file elife-77999-fig4-data4.zip › Figure4sourceData4.Tif]

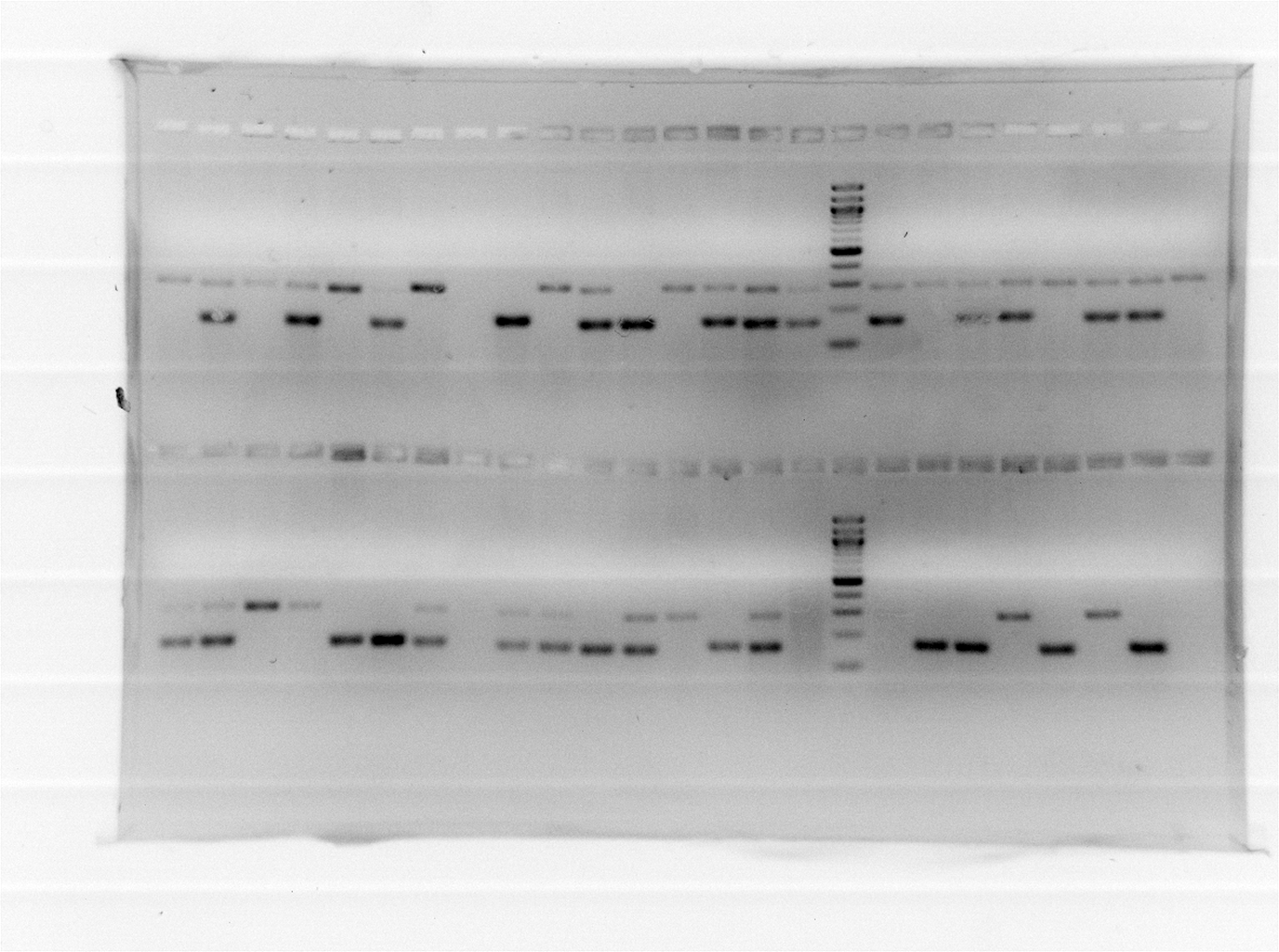

Supplement: Figure 4—source data 5. [file elife-77999-fig4-data5.zip › Figure4sourceData5.Tif]

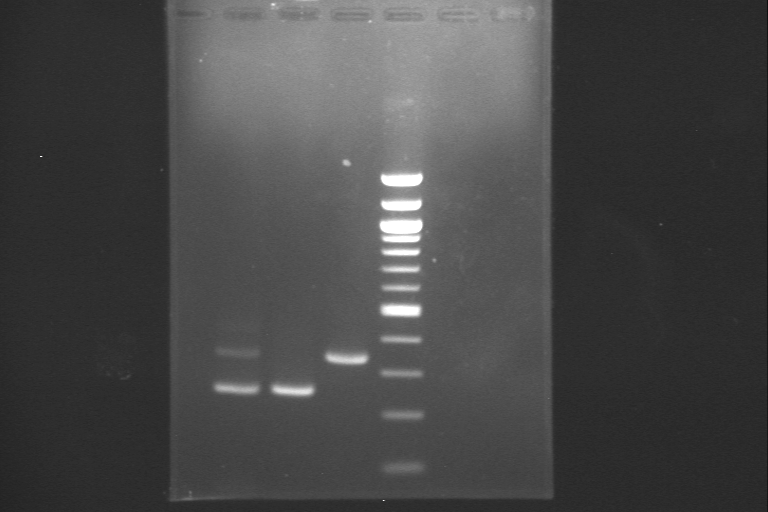

Supplement: Figure 4—source data 6. [file elife-77999-fig4-data6.tif]
