# Supplementary material for: Vision-related convergent gene losses reveal SERPINE3’s unknown role in the eye
Source: eLife. 2022 Jun 21;11:e77999. doi: 10.7554/eLife.77999 (PMC9355568; doi:10.7554/eLife.77999)
Supplement: Transparent reporting form [file elife-77999-transrepform1.docx]

***eLife’s* transparent reporting form**

We encourage authors to provide detailed information *within their submission* to facilitate the interpretation and replication of experiments. Authors can upload supporting documentation to indicate the use of appropriate reporting guidelines for health-related research (see [EQUATOR Network](http://www.equator-network.org/%20)), life science research (see the [BioSharing Information Resource](https://biosharing.org/)), or the [ARRIVE guidelines](http://www.plosbiology.org/article/info:doi/10.1371/journal.pbio.1000412) for reporting work involving animal research. Where applicable, authors should refer to any relevant reporting standards documents in this form.

If you have any questions, please consult our Journal Policies and/or contact us: [editorial@elifesciences.org](mailto:editorial@elifesciences.org).

**Sample-size estimation**

- You should state whether an appropriate sample size was computed when the study was being designed
- You should state the statistical method of sample size computation and any required assumptions
- If no explicit power analysis was used, you should describe how you decided what sample (replicate) size (number) to use

Please outline where this information can be found within the submission (e.g., sections or figure legends), or explain why this information doesn’t apply to your submission:

As severity and penetrance of the *SERPINE3* KO phenotype was unknown, power analysis was not conducted for the KO experiments. Sample size was decided based on practical issues (genotyping is performed on 96 well plates), so we genotyped ~100 fish per generation, expecting ~25 WT and ~25 homozygote individuals for phenotyping (about 10 individuals per gender).

**Replicates**

- You should report how often each experiment was performed
- You should include a definition of biological versus technical replication
- The data obtained should be provided and sufficient information should be provided to indicate the number of independent biological and/or technical replicates
- If you encountered any outliers, you should describe how these were handled
- Criteria for exclusion/inclusion of data should be clearly stated
- High-throughput sequence data should be uploaded before submission, with a private link for reviewers provided (these are available from both GEO and ArrayExpress)

Please outline where this information can be found within the submission (e.g., sections or figure legends), or explain why this information doesn’t apply to your submission:

Replicate numbers are stated in the legends of Figures 3 and 4 and color-coded in Figure 3 and 4. Exclusion/inclusion criteria and handling of outliers are detailed in the Methods within the sections “Forward Genomics Screen”, “Expression analysis of RT-qPCR” and “Microscopy, image processing and analysis”. Raw data of technical replicates, means, median, minimum, maximum, 1^st^ and 3^rd^ quartiles of biological replicates are provided in Figure 3 – source data 1-4 and Figure 4 – source data 1 (qPCRs) as well as in Figure 4 – source data 2 (iris phenotype analysis). For the screen, we provide a supplementary figure (Figure 1 – Figure supplement 1), which details the filtering process.

**Statistical reporting**

- Statistical analysis methods should be described and justified
- Raw data should be presented in figures whenever informative to do so (typically when N per group is less than 10)
- For each experiment, you should identify the statistical tests used, exact values of N, definitions of center, methods of multiple test correction, and dispersion and precision measures (e.g., mean, median, SD, SEM, confidence intervals; and, for the major substantive results, a measure of effect size (e.g., Pearson's r, Cohen's d)
- Report exact p-values wherever possible alongside the summary statistics and 95% confidence intervals. These should be reported for all key questions and not only when the p-value is less than 0.05.

Please outline where this information can be found within the submission (e.g., sections or figure legends), or explain why this information doesn’t apply to your submission:

Statistical tests are specified in legends of Figures 1, 3 and 4 as well as in the methods sections with the following subtitles: “Forward genomics screen”, “Enrichment analysis”, “Selection analysis”, “Expression analysis by RT-qPCR” and “Microscopy, image processing and analysis”. Test details, summary statistics, confidence intervals and measures of effect sizes are provided in Figure 3 – source data 1-4, Figure 4 – source data 1 as well as in Figure 4 – source data 2. For all screens (three different visual acuity thresholds, negative control screen and screen based on molecular loss pattern), we provide the complete list of genes tested along with their p-values, corrected p-values and filtering steps passed in Figure 1 – source data 2 and 4-7. Test details for the GO enrichment analysis are provided in Figure 1 – source data 3 and 9. Test details for the selection test are provided in Figure 2 – source data 4. Raw images of analyzed fish eyes are provided as Figure 4 – source data 7.

(For large datasets, or papers with a very large number of statistical tests, you may upload a single table file with tests, Ns, etc., with reference to sections in the manuscript.)

**Group allocation**

- Indicate how samples were allocated into experimental groups (in the case of clinical studies, please specify allocation to treatment method); if randomization was used, please also state if restricted randomization was applied
- Indicate if masking was used during group allocation, data collection and/or data analysis

Please outline where this information can be found within the submission (e.g., sections or figure legends), or explain why this information doesn’t apply to your submission:

not applicable (group allocation followed the genotype)

**Additional data files (“source data”)**

- We encourage you to upload relevant additional data files, such as numerical data that are represented as a graph in a figure, or as a summary table
- Where provided, these should be in the most useful format, and they can be uploaded as “Source data” files linked to a main figure or table
- Include model definition files including the full list of parameters used
- Include code used for data analysis (e.g., R, MatLab)
- Avoid stating that data files are “available upon request”

Please indicate the figures or tables for which source data files have been provided:

Figure 1 A, B - data in Figure 1 – source data 1, 2

Figure 1 C - data in Figure 1 – source data 3
Figure 2 - data in Figure 2 – source data 1, 4

Figure 3 A - data in Figure 3 – source data 1

Figure 3 B - data in Figure 3 – source data 2

Figure 3 C - data in Figure 3 – source data 3

Figure 3 D - data in Figure 3 – source data 4

Figure 4 A - data in Figure 4 - source data 5-7, figure supplement 2

Figure 4 B - data in Figure 4 - source data 1

Figure 4 E - data in Figure 4 - source data 2

Figure 4 D,E - data in Figure 4 - source data 3
